# Design and experiment of a shovel-tooth removal end-effector for abnormal plants in hybrid rape breeding based on MBD-DEM coupling

**DOI:** 10.1371/journal.pone.0294919

**Published:** 2023-12-14

**Authors:** Qinsong Xing, Suming Ding, Xinyu Xue, Longfei Cui, Feixiang Le, Baokun Wang

**Affiliations:** Nanjing Institute of Agricultural Mechanization, Ministry of Agriculture and Rural Affairs, Nanjing, China; National Kaohsiung University of Science and Technology / Industrial University of Ho Chi Minh, TAIWAN

## Abstract

In view of the problem that removing abnormal plants in breeding rape requires a large amount of labor and is inefficient, combined with the planting requirements of breeding rape, a shovel-tooth end-effector was designed, and a shovel-tooth removal test bench was built. A simulation model based on MBD (Multibody Dynamics)-DEM (Discrete Element Method) coupling was constructed. Then we conducted a Box-Behnken test with four factors and three levels. Taking the angle of soil penetration, speed of soil penetration, depth of soil penetration and speed of shovel-tooth gathering as the test factors, the soil penetration force and shovel-tooth gathering force as the test indicators. The mathematical regression model between test indicators and test factor was established. After optimizing the parameters of the model, the best combination of parameters with low soil penetration force and low shovel-tooth gathering force was obtained: angle of soil penetration of 84°, speed of soil penetration of 9 cm/s, depth of soil penetration of 8cm, and speed of shovel-tooth gathering of 6 cm/s. The simulation model was validated by field experiments. The average soil penetration force and average shovel-tooth gathering force of the three groups of pull-out tests were 34.8 N and 763.0 N, respectively. The removal rates were 96%, 92%, and 94%, all greater than 90%, indicating that the removal effect of the shovel-tooth end-effector was good, and the parameters were reasonably designed. The results can serve as reference for the design of rape abnormal plants removal device and the operation of MBD-DEM coupling simulation end-effector.

## Introduction

Rape, also known as canola, is a globally significant cash crop for oil production and serves as a raw material in the food and other industries [1–4]. The hybridization of rape exhibits evident heterosis in terms of yield, traits, and other relevant aspects. [5, 6]. The annual planting area of Chinese rape is about 7 million hectares, among which the planting area of hybrid rape has accounted for more than 45% [7], and the amount of seed needed is increasing. However, the management of rape breeding field is difficult, especially in the process of removing abnormal plants, which requires a large amount of labor. Ensuring purity is an important link in seed production of hybrid rape, and is the main factor for seed production quality to reach the standard, so it is very important to remove impurities and preserve purity in the breeding process. If the female parent contains 1% abnormal plants, the seed purity will decrease by 4%; if the male parent contains 1% abnormal plants, the hybrid purity will decrease by 1%; if the hybrid purity decreases by 1%, the yield will decrease by 0.2%~0.4% [8]. At present, artificial removal of abnormal plants is the main method in rape breeding fields at home and abroad, and the mechanization operation is basically non-existent. It completely stays at the level of large-scale manual operation, which will consume a lot of manpower, material resources, financial resources and be inefficient. In light of this situation, the research on an automatic and mechanized device for removing abnormal plants holds crucial practical significance for enhancing production efficiency, improving large-scale operational capacity, and reducing production costs.

In the rape breeding field, the abnormal plants should be uprooted and destroyed before the initial flowering stage as much as possible to prevent the residual rhizomes from re-rooting and secondary pollination. Currently, scholars at home and abroad have done a lot of studies on mechanical properties, cutting principles and key components of crop roots [9, 10]. Chen et al. [11] proposed a novel linkage occlusion-cutting mechanism and studied the influence of tool parameters on root cutting force to find the optimal cutting force of garlic root. Zhang et al. [12] designed a bionic root cutter and conducted a test on the cooperation between the root cutter and the ditching device. Jia et al. [13] designed a corn in-row weeding device, which carried out spiral motion between seedlings to eliminate weeds in the row. Machleb et al. [14] designed a new finger weeding manipulator equipped with electric motors based on the traditional finger weeding manipulator. The finger weeding manipulator constantly broke the root soil for weeding. After analyzing the aforementioned literature, it can be found that most of the devices studied above are aimed at crop root cutting and soil crushing. However, no research has been reported on the removal device of abnormal plants in rape breeding field.

Most research methods of traditional agricultural machinery are predominantly grounded in theoretical analysis and experimental research [15]. However, the latter approach is characterized by its time-consuming and labor-intensive nature, along with significant limitations. In recent years, with the rapid development of computer technology, DEM-MBD coupling numerical simulation technology has been widely used in agricultural engineering field [16–18]. Richter et al. [19] proposed a program-based DEM-MBD coupling method and verified the superiority of the simulation results. Mohajeri et al. [20] established a sequential multi-objective optimization framework for the grab bucket of ship unloader, and adopted the DEM-MBD coupling simulation method to verify the optimization results. Based on DEM-MBD coupling technology, Wang et al. [21] established a picker simulation model with straw as the research object and verified the accuracy of the simulation model. The above research showcases the reliability of the coupling simulation method, which has unique advantages of saving time and effort. In this paper, MBD-DEM coupling simulation method is used for simulating the shovel-tooth end-effector, which can not only observe the interaction between equipment and soil more intuitively, but also provide a reference for the next structural optimization.

In summary, there have been no reports on the research of devices for removing abnormal rape plants. This paper designs a shovel-tooth end-effector for this situation. A simulation model was developed based on the coupling of Adams-EDEM, utilizing the Multi-Body Dynamics (MBD) and Discrete Element Method (DEM) coupling techniques to accurately simulate the process of removing abnormal plants in field conditions. Quadratic regression orthogonal rotation combination simulation experiments were conducted to obtain the optimal operation parameters of the end-effector. And a test bench was built for field testing and verification, in order to provide reference for the design of rape abnormal plants removal devices for breeding.

## Materials and methods

### Entire structure and the working principle of test bench

#### The dimensions of entire structure

**Fig 1:**
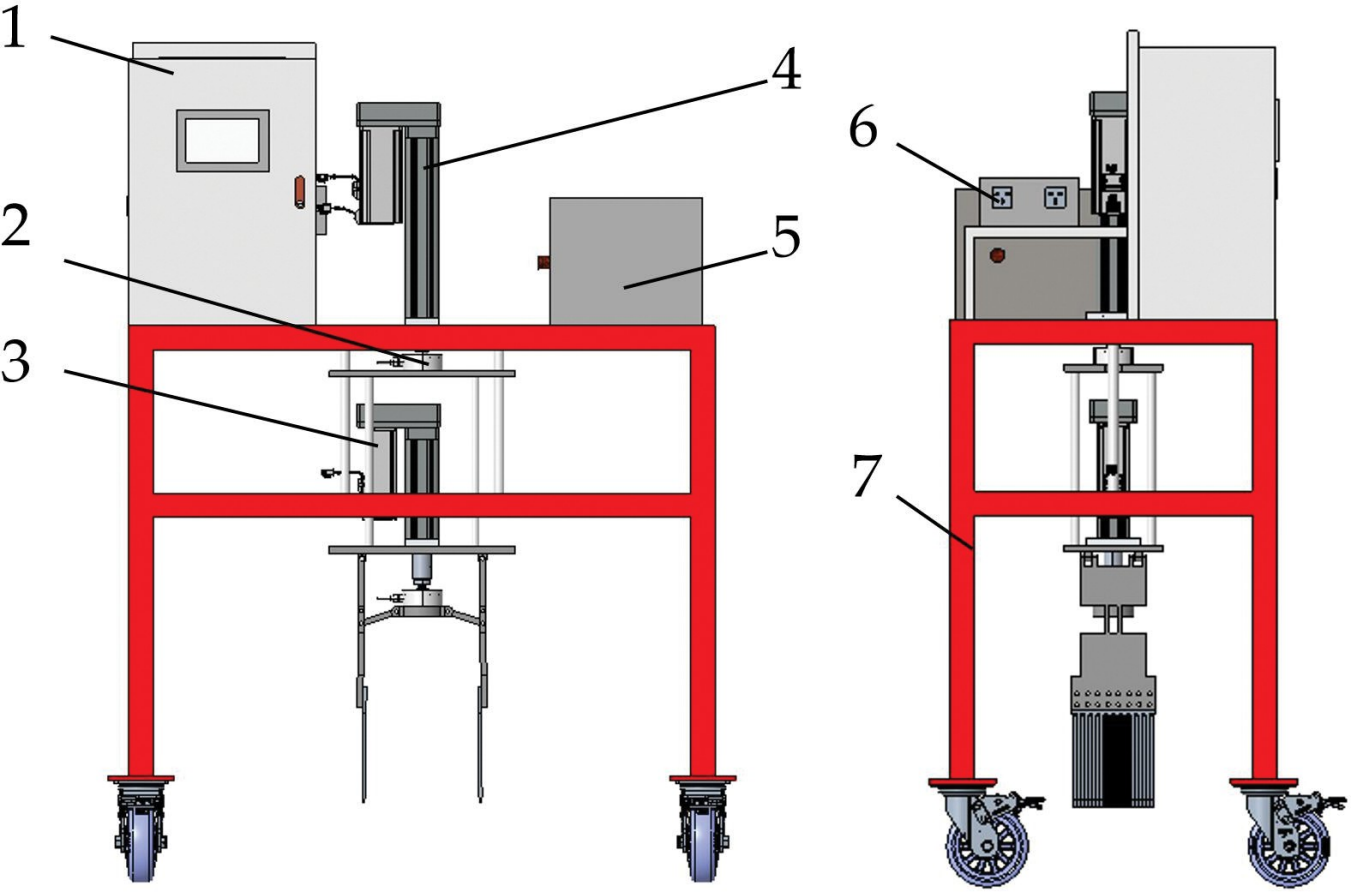
Entire structure of test bench: 1. control cabinet; 2. test bench tension pressure sensor; 3. end-effector; 4. test bench servo electric cylinder; 5. battery; 6. inverter; 7. mobile bench.

The end-effector requires a test platform that can easily be used with the end-effector for testing operations. Firstly, the platform needs to be moved to the specified point, and secondly, it should be equipped with an electrical control system that allows the end-effector to work. According to the requirement of end-effector to pull out rape abnormal plants, a shovel-tooth removal test bench was designed, which was mainly composed of control cabinet, test bench tension pressure sensor, end-effector, test bench servo electric cylinder, battery, inverter and mobile bench. The overall dimension of the test bed is 1100mm×600mm×1400mm, and the basic structure is shown in Fig 1.

#### Working principle of test bench

**Fig 2:**
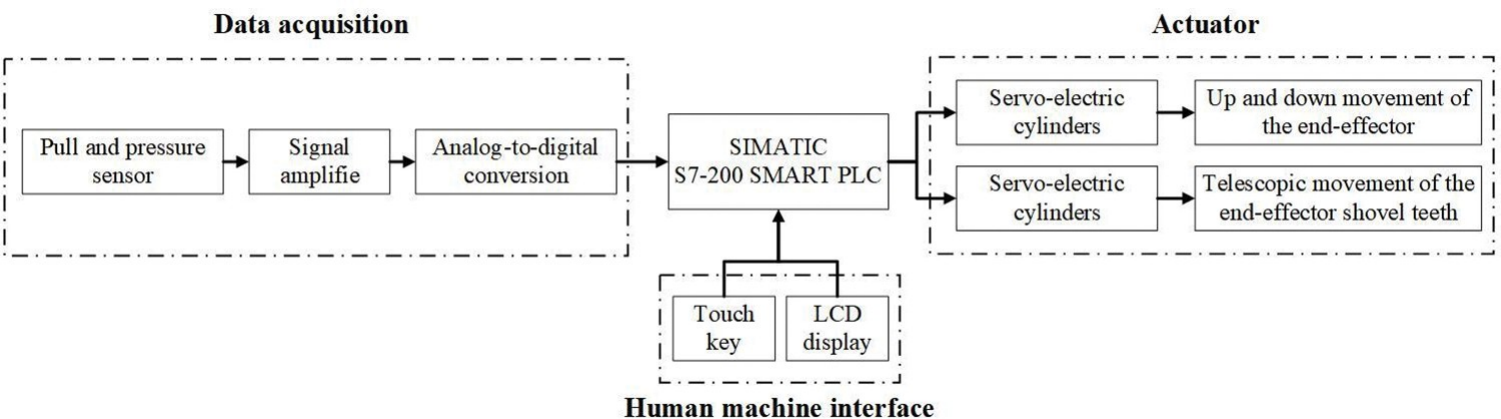
Schematic diagram of test bench.

The test bench is capable of simulate the removal process and mode of abnormal plants in breeding rape fields, while also enabling the collection of the force of shovel-tooth. The working principle is shown in Fig 2. During the field test, the distance and position of abnormal plants and the end-effector can be adjusted by moving the bench. Different test parameters can be specified through the human-machine interaction interface on the control cabinet according to the test requirements, including the angle of soil penetration, speed of soil penetration, depth of soil penetration and speed of shovel-tooth gathering. The human-machine interaction interface transfers the specified experimental parameters input to the PLC through the RS485 serial port. Then the PLC controls the servo electric cylinder to drive the end-effector to move according to the specified test parameters. During the system operation, the test bench can collect and store data.

### Design of end-effector

**Fig 3:**
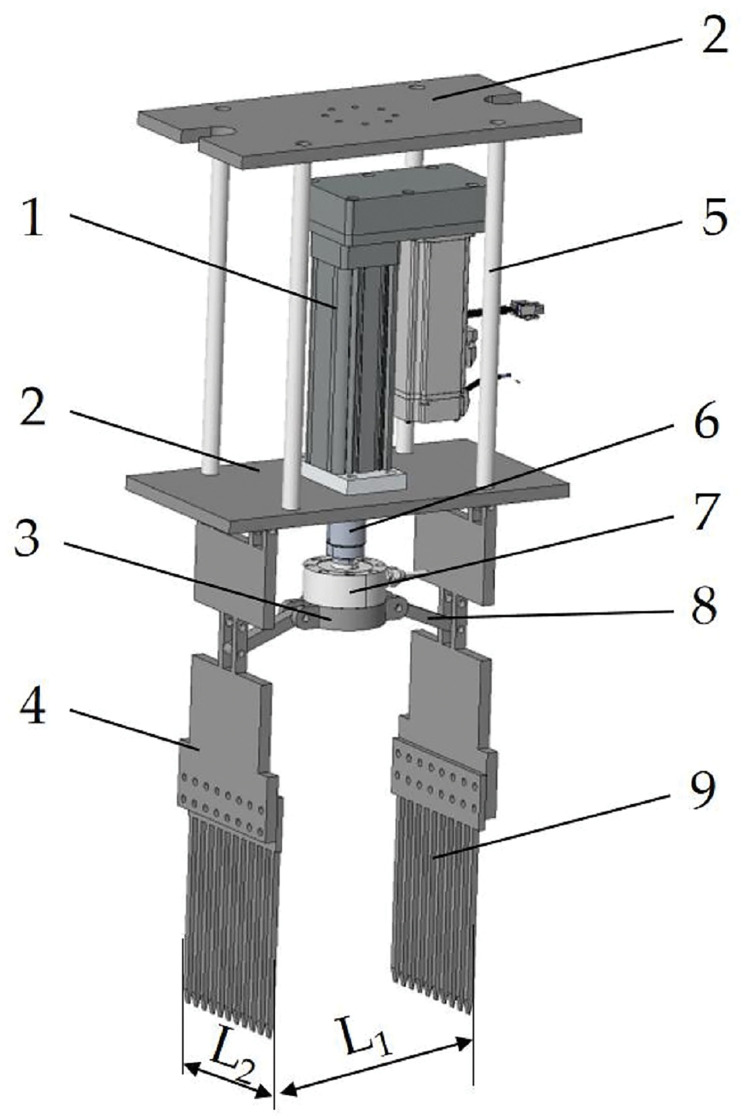
Structure of test bench: 1. end-effector servo electric cylinder; 2. support plate; 3. connecting rod connector; 4. shovel-tooth plate; 5. support rod; 6. Electric Pushing Rod; 7. end-effector tension pressure sensor; 8. connecting rod; 9. shovel-tooth.

The end-effector is one of the important working components of the test bench, which is mainly used for removing abnormal plants. The structure is shown in Fig 3, which is mainly composed of the end-effector servo electric cylinder, support plate, connecting rod, end-effector tension pressure sensor, shovel-tooth, etc. The plant spacing of rape breeding field is 300 mm and the row spacing is 350 mm. The shovel-tooth end-effector is utilized for excavating soil and extracting aberrant plants from the field, while minimizing disruption to the root systems of healthy vegetation. Therefore, considering the growth of rape at the initial flowering stage and the requirements of planting and agronomy, the distance L_1_ between the shovel-tooth and the shovel-tooth is designed to be 220 mm, and the width L_2_ of the shovel-tooth is 152 mm. The shovel-tooth is fixed on the shovel-tooth plate by bolts, and the two are connected to each other through a connecting rod connector. The connecting rod connector is equipped with the end-effector tension pressure sensor, which is connected to the end-effector servo electric cylinder. The end-effector support plate is connected with the test bench tension pressure sensor, which is fixed on the push rod of the test bench servo electric cylinder. The test bench servo electric cylinder can provide the power for the end-effector to move.

Upon receiving the specified test parameters from the human-machine interface, the programmable logic controller (PLC) transmits motion instructions to activate the two servo-electric cylinders in the actuator. Firstly, the test bench servo electric cylinder drives the end-effector to enter the soil. Then the end-effector servo electric cylinder drives the shovel-tooth plate and shovel-tooth to gather. Finally the test bench servo electric cylinder drives the end-effector to leave the soil. At this time, the shovel-tooth can pull out the abnormal plants and the soil around the root of the abnormal plants.

### Simulation test of end-effector operation

The interaction between shovel-tooth and connecting rod involves multi-body kinematics, and the interaction between shovel-tooth and soil involves multi-body kinematics and discrete element theory. Therefore, MBD-DEM coupling method is used for the analyses. This paper used the Adams-EDEM coupling simulation method to simulate the operational state of the shovel-tooth end-effector. On this basis, a simulation model of the end-effector and soil was established to simulate the interaction between end-effector and soil during the operation of the test bench.

#### Construction of the MBD model

Adams software is capable of simulating the motion of end-effector, and the motion characteristics can be analyzed through its post-processing interface.

The end-effector model was established and assembled in Creo 3D software, exported as step format, and then imported into Adams View environment for dynamic simulation. The component material was set to be steel, forming the center of mass. The model was simplified and the fasteners were removed to reduce the number of unnecessary constraints in the simulation process to improve the simulation efficiency. Constraints needed to be added to each mechanism, and the constraint Settings for each component were shown in Table 1. A moving drive was added between the support plate and the earth to simulate the process of end-effector insertion and excavation. A moving drive was added between the end-effector servo electric cylinder and the push rod on the end actuator servo electric cylinder. By moving the push rod of the electric cylinder up and down, the folding and stretching motion of the shovel-tooth can be driven by the connecting rod. A moving drive was added between the end-effector servo electric cylinder and the electric push rod, and the motion of the shovel-tooth could be driven by the up and down movement of the push rod of the electric cylinder.

**Table 1:** Kinematic pair configuration table of multi-body dynamics model.

No.	Part 1	Part 2	Kinematic pair
1	Support plant	Earth	Moving pair
2	End-effector servo electric cylinder	Support plant	Fixed pair
3	Electric push rod	End-effector servo electric cylinder	Moving pair
4	Connecting rod connector	Push rod	Fixed pair
5	Connecting rod	Connecting rod connector	Rotating pair
6	Shovel-tooth plate	Support plant	Rotating pair
7	Connecting rod	Shovel-tooth plate	Rotating pair
8	Shovel-tooth	Shovel-tooth plate	Fixed pair

#### Construction of the DEM Model

The 3D model of the shovel-tooth end-effector was imported into EDEM. The soil particle bed, measuring 800mm×600mm×300mm, was established 100mm below the end-effector equipped with a shovel-tooth. All particles were single ball particles with a size of 8mm. Because the adhesion force and friction force between rape roots and soil were much smaller than the force of soil extrusion, the discrete element model was simplified and the rape plant model is not established.

In order to better simulate the interaction between end-effector and soil, it is necessary to select an appropriate discrete element contact model and determine appropriate model parameters [22–24]. Researchers at the University of Edinburgh proposed an Edinburgh Elasto-Plastic Adhesion (EEPA) model. This model included the plasticity and viscosity of soil particles, making it suitable for simulating soils with strong plasticity. Hertz-Mindlin with JKR contact model was a cohesive contact model suitable for simulating wet viscous soil. This model was consistent with the effect of end-effector on soil moisture and viscosity during operation. In this paper, the EEPA model was selected as the soil-soil contact model, and the Hertz-Mindlin with JKR model was selected as the soil-end-effector contact model. By consulting the literature [25], we determined the contact parameters and fundamental physical parameters, as shown in Table 2.

**Table 2:** Parameter configuration table of discrete element for soil.

No.	Part 1	Part 2
1	Soil particle radius /mm	8
2	Poisson’s ratio of the soil	0.38
3	Soil particle density /(kg·m^-3^)	2600
4	Shear modulus between the soil / MPa	1
5	Coefficient of Restitution between the soil	0.37
6	Coefficient of Static Friction between the soil	0.6
7	Coefficient of Rolling Friction between the soil	0.26
8	Adhesion strength between the soil /N	-0.001
9	Contact surface energy between the soil particles /J·m^2^	15.6
10	Contact plasticity ratio between the soil	0.36
11	Coefficient of Restitution between soil and shovel-tooth	0.54
12	Coefficient of Static Friction between soil and shovel-tooth	0.31
13	Coefficient of Rolling Friction between soil and shovel-tooth	0.13

#### MBD-DEM coupling simulation

**Fig 4:**
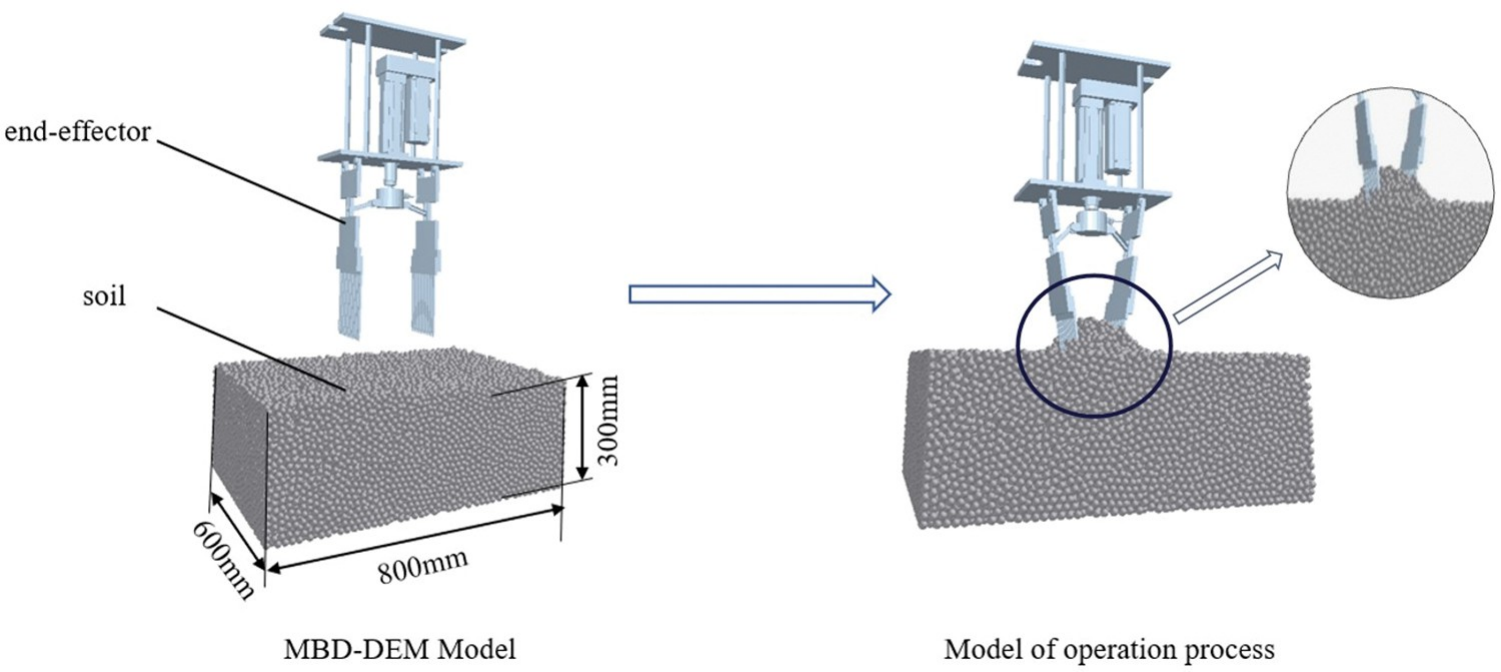
The construction of MBD-DEM coupling simulation model.

During the MBD-DEM coupling simulation test, the randomness of the simulation test was reflected by regenerating the soil particle bed before each test. Configure the *.acf file and *.cosim file while ensuring that the coupling interface between Adams and EDEM was open. Output the *.adm file through Adams and modify the environment variables in the *.adm file. Load *.cosim file in Adams Co-simulation to realize two-way real-time data transfer of Adams-EDEM. The time step of EDEM was 0.0004 s, and the target save time was 0.01 s. The DEM-MBD coupling simulation model was established, as shown in Fig 4.

### Orthogonal rotation combination design test

A four-factor quadratic regression general rotary combination test was designed to optimize the key parameters of the end-effector operation. Taking the soil penetration force(Y_1_) and the shovel-tooth gathering force(Y_2_) as the response values, the response surface test was carried out on the angle of soil penetration(X_1_), speed of soil penetration(X_2_), depth of soil penetration(X_3_) and speed of shovel-tooth gathering(X_4_). Combined with the structure of the shovel-tooth end-effector, the angle of soil penetration range was determined to be 84° ~ 90°. The speed of soil penetration range was determined to be 8 cm/s ~ 12 cm/s. The root length of rape before the initial flowering stage is generally less than 10cm, so the depth of soil penetration range was determined to be 10 cm ~ 14 cm. The speed of shovel-tooth gathering range was determined to be 6 cm/s ~ 10 cm/s. Test factors and levels are shown in Table 3.

**Table 3:** Parameter configuration table of discrete element for soil.

Levels	Factors			
	Angle of soil penetration *X*_1_ /°	Speed of soil penetration *X*_2_ /(cm·s^-1^)	Depth of soil penetration *X*_3_ /cm	Speed of shovel-tooth gathering *X*_4_ /(cm·s^-1^)
-1	84	8	10	6
0	87	10	12	8
1	90	12	14	10

The test program was derived based on Box-Behnken principle, and there were 29 groups of tests. The data analysis of this experiment was conducted using Design-Expert software to establish a quadratic polynomial regression model and perform variance analysis on the regression equation. The 3D corresponding surface was established to analyze the interaction between each factor. Through the above analysis, we optimized the experimental results, which were subsequently validated through a field experiment.

## Results

### The result of orthogonal rotation combination design test

#### Establishment of the regression model and analysis of variance

At the conclusion of the experiment, the simulation results were imported into the Adams post-processing interface, and the data with large fluctuations were eliminated. The Design-Expert software was used for data fitting analysis. Taking the maximum data of the soil penetration force(Y_1_) and the shovel-tooth gathering force(Y_2_) as the corresponding index results, and taking the soil penetration force(Y_1_) and the shovel-tooth gathering force(Y_2_) as the dependent variables, multiple regression equations of the angle of soil penetration(X_1_), speed of soil penetration(X_2_), depth of soil penetration(X_3_) and speed of shovel-tooth gathering(X_4_) were established. The results of the experiment are shown in Table 4.


$$Y_{1}=54.57+5.45X_{1}+3.95X_{2}+8.82X_{3}+0.04X_{4}‐2.04X_{1}X_{2}+1.78X_{1}X_{3}+0.35X_{1}X_{4}+0.51X_{2}X_{3}‐0.02X_{2}X_{4}+0.09X_{3}X_{4}‐1.04X_{1}{}^{2}‐1.78X_{2}{}^{2}‐3.76X_{3}{}^{2}‐0.8X_{4}{}^{2}$$ (1)

$$Y_{2}=938.88+104.29X_{1}‐4.85X_{2}+142.85X_{3}+23.2X_{4}‐X_{1}X_{2}+29.55X_{1}X_{3}‐1.1X_{1}X_{4}‐3.54X_{2}X_{3}‐5.84X_{2}X_{4}‐1.76X_{3}X_{4}+29.07X_{1}{}^{2}+4.1X_{2}{}^{2}+11.61X_{3}{}^{2}+4.33X_{4}{}^{2}$$ (2)

**Table 4:** Experimental program and results.

No.	Angle of soil penetration *X*_1_ /°	Speed of soil penetration *X*_2_ /(cm·s^-1^)	Depth of soil penetration *X*_3_ /cm	Speed of shovel-tooth gathering *X*_4_ /(cm·s^-1^)	Soil penetration force *Y*_1_ /N	Shovel-tooth gathering force *Y*_2_ /N
1	-1	-1	0	0	40.35	871.12
2	1	-1	0	0	55.22	1085.25
3	-1	1	0	0	52.31	865.30
4	1	1	0	0	59.02	1075.42
5	0	0	-1	-1	41.13	802.15
6	0	0	1	-1	58.75	1075.60
7	0	0	-1	1	41.03	842.00
8	0	0	1	1	59.00	1108.41
9	-1	0	0	-1	47.02	850.18
10	1	0	0	-1	59.23	1065.32
11	-1	0	0	1	46.56	889.48
12	1	0	0	1	60.18	1100.20
13	0	-1	-1	0	36.45	828.61
14	0	1	-1	0	44.20	832.18
15	0	-1	1	0	53.89	1092.10
16	0	1	1	0	63.68	1081.50
17	-1	0	-1	0	38.25	737.14
18	1	0	-1	0	43.68	878.74
19	-1	0	1	0	51.34	1008.81
20	1	0	1	0	63.89	1268.60
21	0	-1	0	-1	48.00	911.22
22	0	1	0	-1	55.09	905.13
23	0	-1	0	1	47.95	988.70
24	0	1	0	1	54.95	959.26
25	0	0	0	0	55.32	930.30
26	0	0	0	0	54.90	932.00
27	0	0	0	0	56.20	962.68
28	0	0	0	0	55.23	943.40
29	0	0	0	0	51.20	926.00

The variance analysis and significance test of the regression model are presented in Table 5. The regression models of soil penetration force(*Y*_1_) and shovel-tooth gathering force(*Y*_2_) were extremely significant (*P*<0.01), and the lack of fit was not significant (*P*>0.05). These results indicated that the regression equation demonstrates a strong degree of fitting, enabling analysis and optimization of key parameters related to end-effector operation.

**Table 5:** Variance analysis of regression equations.

Source	Soil penetration force *Y*_1_/N					Shovel-tooth gathering force *Y*_2_ /N			
	Sum of Squares	df	*F* Value	*p*-Value		Sum of Squares	df	*F* Value	*p*-Value
Model	1606.76	14	53.32	< 0.0001 **		3.916E+05	14	74.52	< 0.0001 **
*X*_1_	356.32	1	165.54	< 0.0001 **		1.305E+05	1	347.67	< 0.0001 **
*X*_2_	187.15	1	86.94	< 0.0001 **		282.37	1	0.7522	0.4004
*X*_3_	932.98	1	433.43	< 0.0001 **		2.449E+05	1	652.28	< 0.0001 **
*X*_4_	0.0169	1	0.0078	0.9307		6461.20	1	17.21	0.0010 **
*X*_1_ *X*_2_	16.65	1	7.73	0.0147 *		4.02	1	0.0107	0.9190
*X*_1_ *X*_3_	12.67	1	5.89	0.0293 *		3492.22	1	9.30	0.0087 **
*X*_1_ *X*_4_	0.497	1	0.2309	0.6383		4.88	1	0.0130	0.9108
*X*_2_ *X*_3_	1.04	1	0.4833	0.4983		50.20	1	0.1337	0.7201
*X*_2_ *X*_4_	0.0020	1	0.0009	0.9760		136.31	1	0.3631	0.5564
*X*_3_ *X*_4_	0.0306	1	0.0142	0.9067		12.39	1	0.0330	0.8584
*X*_1_^2^	6.96	1	3.23	0.0937		5482.57	1	14.60	0.0019 **
*X*_2_^2^	20.51	1	9.53	0.0080 **		109.12	1	0.2907	0.5983
*X*_3_^2^	91.50	1	42.51	< 0.0001 **		874.00	1	2.33	0.1493
*X*_4_^2^	4.21	1	1.96	0.1836		121.84	1	0.3246	0.5779
Residual	30.14	14				5255.77	14		
Lack of Fit	15.01	10	0.3972	0.8923		4382.05	10	2.01	0.2622
Pure Error	15.12	4				873.72	4		
Cor Total	1636.90	28				3.969E+05	28		

Note

** expresses highly significant (p ≤ 0.01)

* expresses significant (0.01 ≤ p ≤ 0.05).

The significance of the influence of each regression term on the regression model is determined by the size of the *P* value. In *Y*_1_ regression model, the influence of primary terms *X*_1_, *X*_2_ and *X*_3_ was extremely significant (*P*< 0.01). The interaction terms *X*_1_*X*_2_ and *X*_1_*X*_3_ had a significant effect (*P*<0.05). The influence of square terms *X*_2_^2^ and *X*_3_^2^ was extremely significant (*P*<0.01). *Y*_2_ was significantly affected by *X*_1_, *X*_2_, *X*_3_, interaction term *X*_1_*X*_3_ and square terms *X*_1_^2^ (*P*<0.01). On the premise of ensuring that the model was significant and the lack of fit was not significant, the insignificant regression term was eliminated. The regression equation optimization results were as follows.

**Fig 5:**
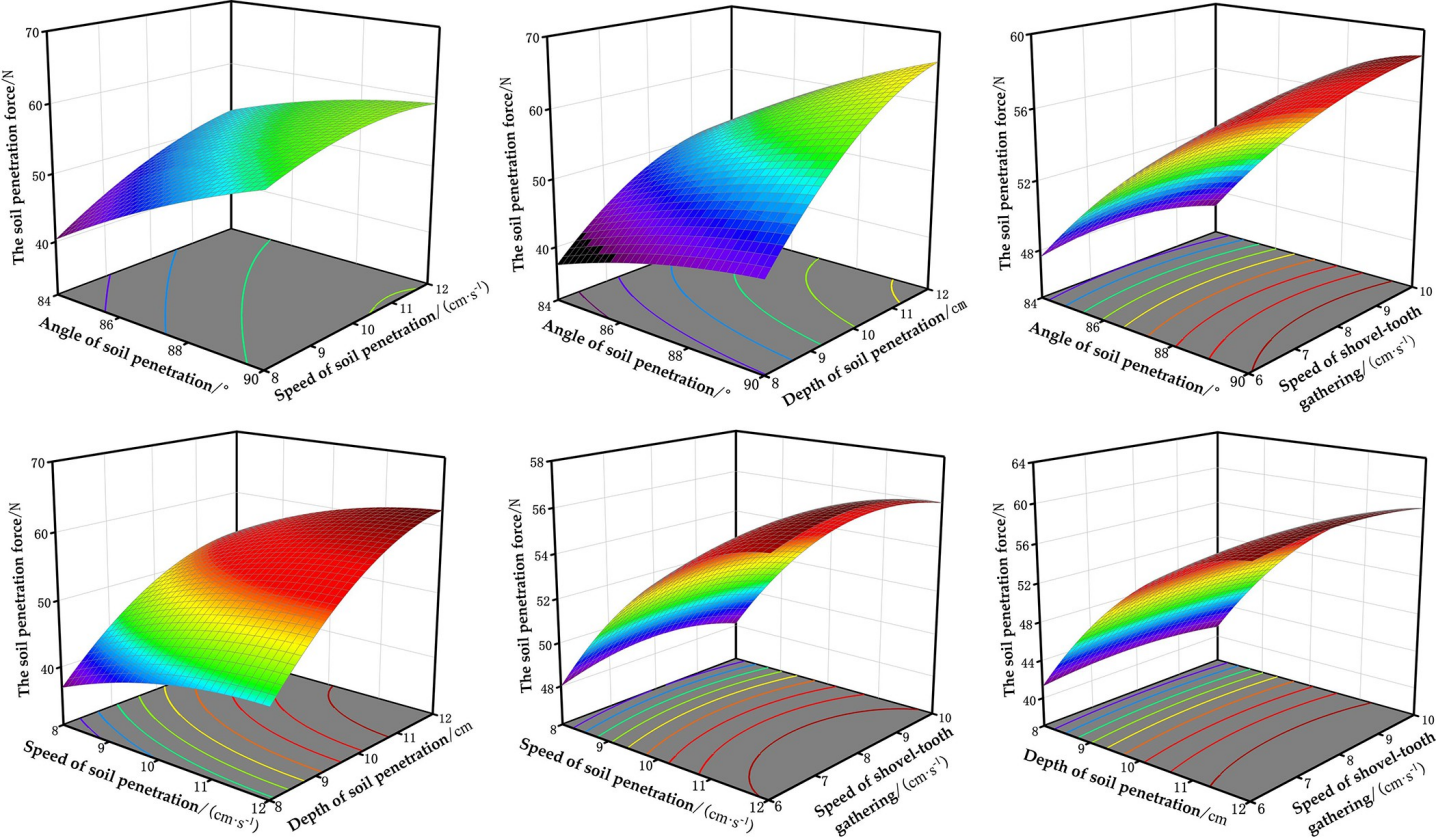
Response surface plots of each factor on the soil penetration force. (a)*Y*_1_ (*X*_1_, *X*_2_, 10, 8). (b)*Y*_1_ (*X*_1_, 10, *X*_3_, 8). (c)*Y*_1_ (*X*_1_, 10, 10, *X*_4_). (d)*Y*_1_ (87, *X*_2_, *X*_3_, 8). (e)*Y*_1_ (87, *X*_2_, 10, *X*_4_). (f)*Y*_1_ (87, 10, *X*_3_, *X*_4_).

**Fig 6:**
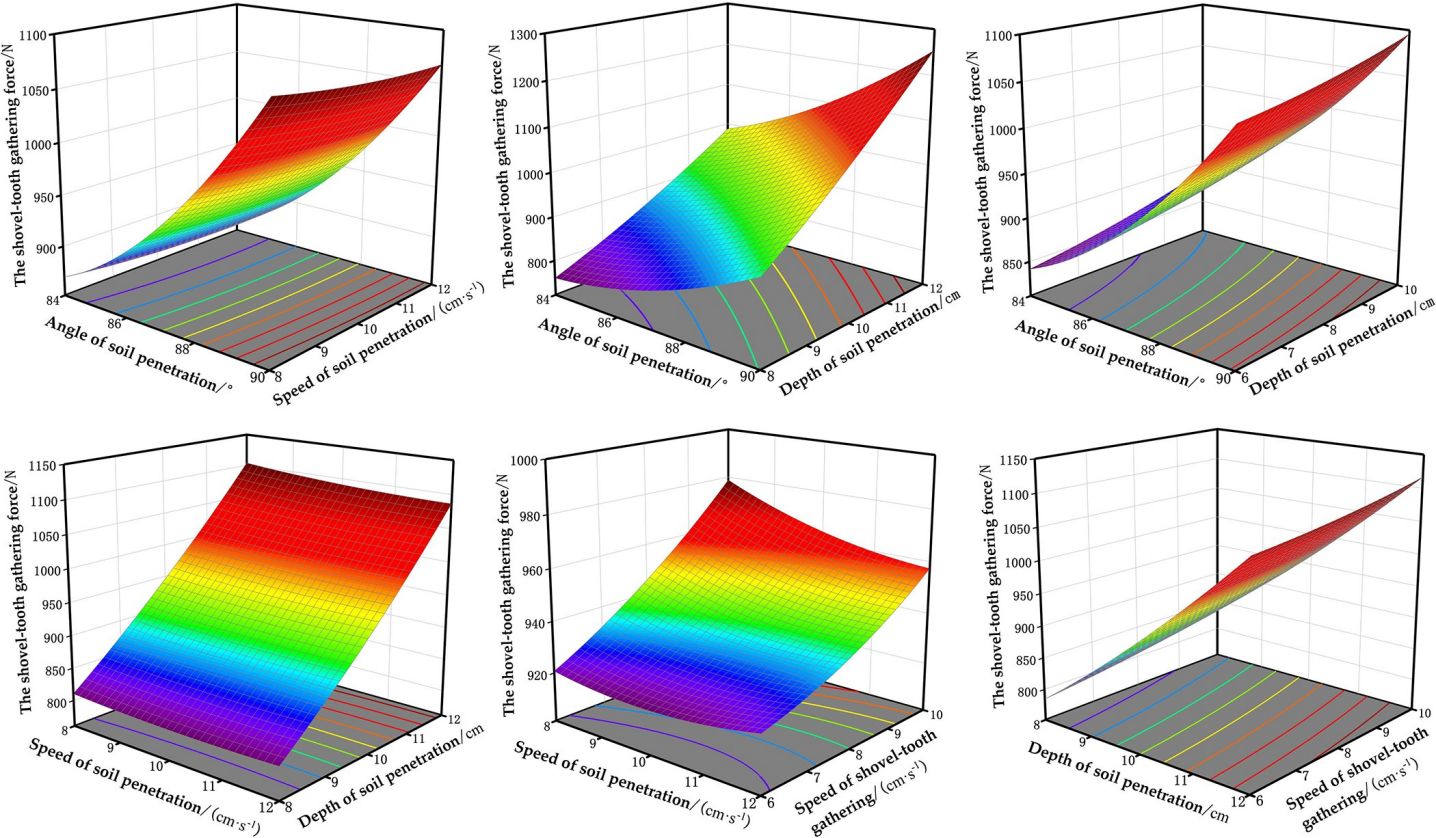
Response surface plots of each factor on the shovel-tooth gathering force. (a)*Y*_2_ (*X*_1_, *X*_2_, 10, 8). (b)*Y*_2_ (*X*_1_, 10, *X*_3_, 8). (c)*Y*_2_ (*X*_1_, 10, 10, *X*_4_). (d)*Y*_2_ (87, *X*_2_, *X*_3_, 8). (e)*Y*_2_ (87, *X*_2_, 10, *X*_4_). (f)*Y*_2_ (87, 10, *X*_3_, *X*_4_).

The effects of the interaction terms on the soil penetration force *Y*_1_ and the shovel-tooth gathering force *Y*_2_ are illustrated in Figs 5 and 6, respectively. The influence of four factors on the soil penetration force *Y*_1_ was in the order of *X*_3_, *X*_1_, *X*_2_ and *X*_4_, among which the interaction of *X*_1_*X*_2_ and *X*_1_*X*_3_ could not be ignored. The results presented in Fig 5(A) demonstrate a positive correlation between the angle of soil penetration and the corresponding soil penetration force, which is further influenced by the speed of soil penetration. The results depicted in Fig 5(B) demonstrate a positive correlation between the angle and depth of soil penetration with the corresponding soil penetration force, indicating that an increase in either parameter leads to a greater exertion of force during soil penetration. The influence of four factors on the shovel-tooth gathering force Y2 was in the order of X3, X1, X4 and X2, among which the interaction of *X*_1_*X*_3_ could not be ignored. Fig 6(B) show that the shovel-tooth gathering force increases with increasing angle of soil penetration, and the changing trend is small at a small depth of soil penetration. The shovel-tooth gathering force increases with increasing depth of soil penetration and the trend is greater when the angle of soil penetration is higher.

As shown in Fig 5A–5F, with the increase of the angle of soil penetration, speed of soil penetration and depth of soil penetration, the soil penetration force increases, while the speed of shovel-tooth gathering has little effect on the soil penetration force. As shown in Fig 6A–6F, with the increase of the angle of soil penetration, depth of soil penetration and speed of shovel-tooth gathering, the shovel-tooth gathering force increases, while the speed of soil penetration has little effect on the shovel-tooth gathering force. In order to minimize the operating power consumption of the end-effector, it is required that the soil penetration force and shovel-tooth gathering force should be as small as possible. Therefore, in order to minimize the soil penetration force, it is advisable to maintain a low angle, speed, and depth of soil penetration, while ensuring a moderate rate of shovel-tooth gathering. In order to reduce the shovel-tooth gathering force, the angle of soil penetration, depth of soil penetration and speed of shovel-tooth gathering should be at a low level, and the speed of soil penetration should be at a moderate level.

#### Parameter optimization

Based on the aforementioned analysis results, in order to reduce the power consumption of the end-effector during operation, it was required to minimize the soil penetration force and shovel-tooth gathering force. Consequently, constraints are derived and mathematical models are built based on the levels of the test factors and the minimum values of the test indicators.


$$\left\{ \begin{array}{c} minY_{1}(X_{1},X_{2},X_{3},X_{4})\\ minY_{2}(X_{1},X_{2},X_{3},X_{4})\\ 84\leq X_{1}\leq 90\\ 8\leq X_{2}\leq 12\\ 8\leq X_{3}\leq 12\\ 6\leq X_{4}\leq 10 \end{array}\right.$$ (3)

Multi-objective optimization was performed using the Numerical component of the Design-Expert software. With the angle of soil penetration of 84°, speed of soil penetration of 9.0 cm/s, depth of soil penetration of 8.0 cm and speed of shovel-tooth gathering of 6.0 cm/s, the soil penetration force *Y*_1_ was predicted to be 33.8 N, and the shovel-tooth gathering force *Y*_2_ was 738.5 N. These parameters were substituted into the MBD-DEM model, and the tests were repeated for three times. The soil penetration force was 33.4 N, 34.1 N and 34N, and the shovel-tooth gathering force was 739.8 N, 736.2 N and 740.4 N.

### Field test verification

#### Test conditions and equipment

The field experiment was carried out according to the optimized parameters, and the test results were recorded. On November 3, 2022, the team conducted a field test on a test bench in Nanjing, Jiangsu Province. The soil conditions of the test field are shown in Table 6.

**Table 6:** Soil conditions.

Project	Technical Parameters
Soil type	Yellow clay
Soil water content/%	23.3
Soil compactness/kPa	275

**Fig 7:**
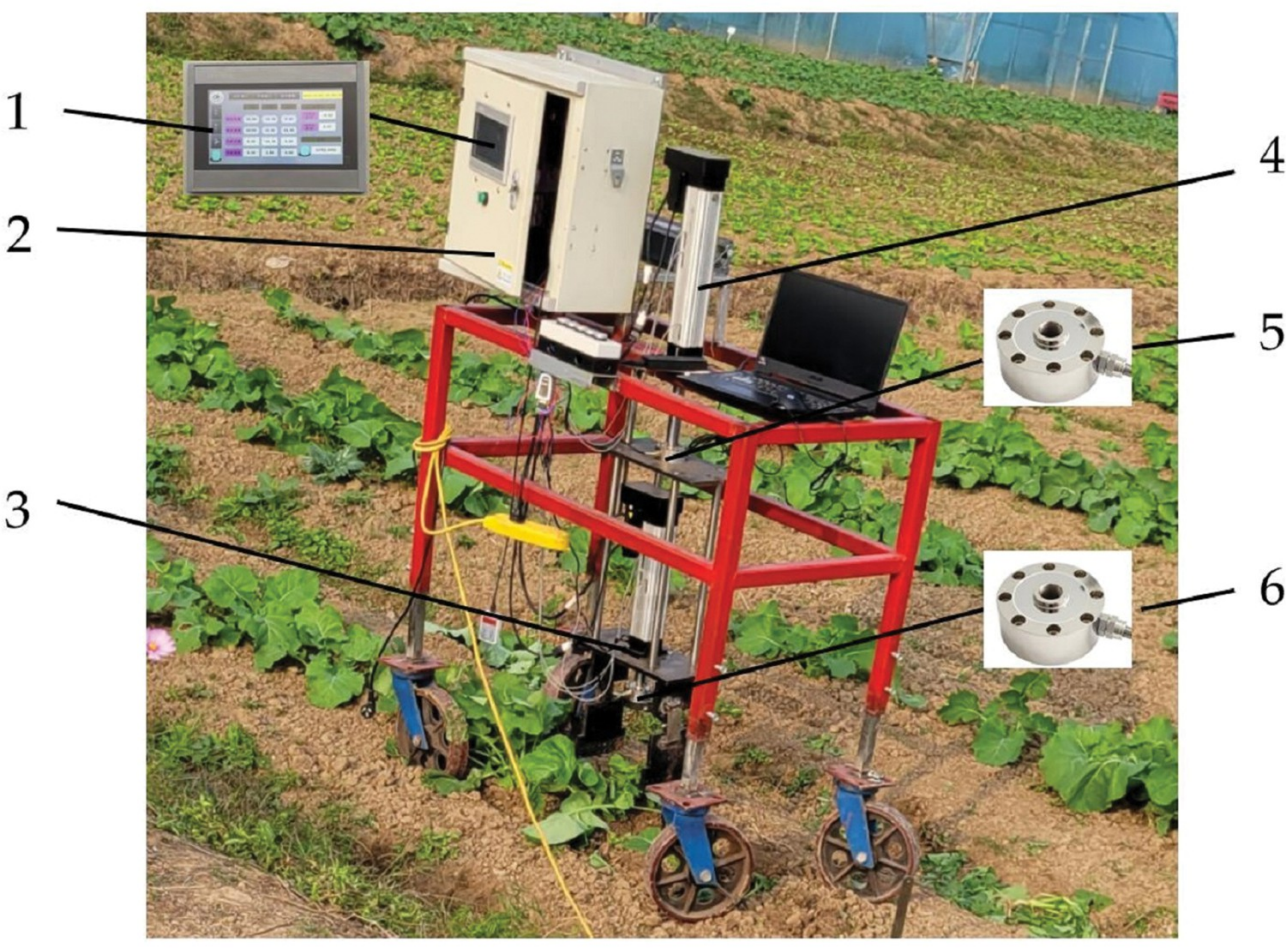
Test equipment: 1. touch screen; 2. control cabinet; 3. end-effector; 4. test bench servo electric cylinder; 5. test bench tension pressure sensor; 6. end-effector tension pressure sensor.

As shown in Fig 7, the test equipment is a shovel-tooth removal test bench, which mainly includes control cabinet, test bench tension pressure sensor, end-effector, test bench servo electric cylinder, etc. The test bench data acquisition system was mainly composed of two digital transmitters (Spartop ABT904 digital transmitter, comprehensive error of 0.01%, output signal 0~10V), test bench tension pressure sensor and end-effector tension pressure sensor (comprehensive error of 0.05%), and configuration touch screen. The tension pressure sensor could convert the variable pressure signal into a corresponding non-standard electrical signal. Because non-standard electrical signals could not be interpreted by PLC, it was necessary to equip a signal transmitter. The non-standard electrical signal was converted into a 0–10 V standard signal by the signal transmitter and transmitted to the PLC via the analog expansion module. The PLC sent the signal processing to the touch screen through the RS485 bus, and then the pressure data was recorded into the USB flash through the touch screen. Before each test, the pressure sensor was set to 0 through the touch screen to ensure the measurement accuracy. The test bench tension pressure sensor was responsible for collecting the soil penetration force, and the end-effector tension pressure sensor was responsible for collecting the shovel-tooth gathering force.

#### Test index and method

The evaluation indexes of field verification test were soil penetration force, shovel-tooth gathering force and removal rate. Based on the actual situation in the field, the angle of soil penetration of 84°, speed of soil penetration of 9.0 cm/s, depth of soil penetration of 8.0 cm and speed of shovel-tooth gathering of 6.0 cm/s were determined for testing. Three groups of removal experiments were carried out on the same land at the same time, and 50 rapes were tested in each group. The soil penetration force, shovel-tooth gathering force and whether to pull out each sample were recorded. Finally, the average soil penetration force, average shovel-tooth gathering force, relative error and removal rate of each group were calculated. The removal rate refers to the ratio of the number of samples successfully removed by the end-effector to the total number of samples. The removal rate and relative error can be expressed as follows.


$$T=W_{1}/W\times 100\%$$ (4)

where, *T* is the removal rate, *W*_1_ is the number of samples successfully removed from the end-effector, *W* is the total number of samples that need to be removed.

$$\delta =|Q1–Q_{2}|/Q_{2}\times 100\%$$ (5)

where, *δ* is the relative error, *Q*_1_ is the test average, *Q*_2_ is the simulation prediction value.

#### Test results and analysis

**Fig 8:**
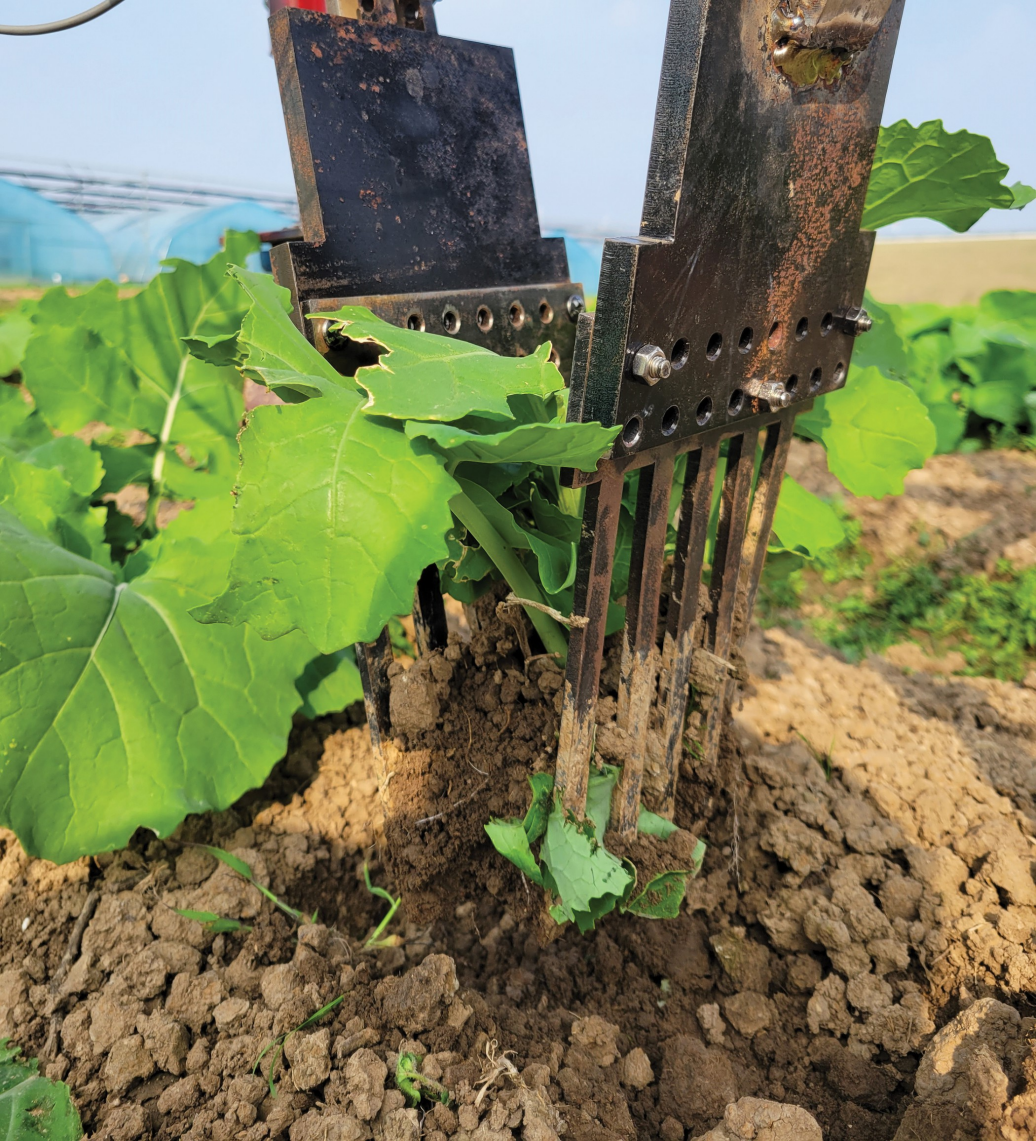
Operation effect picture.

The test results of the three groups of removal tests were presented in Table 7, and the field operation effect of the end-effector was illustrated in Fig 8. It can be seen from Table 7 that the average soil penetration force of the three groups of pullout tests was 34.8 N, the average shovel-tooth gathering force was 763.0 N, and the relative error of force was less than 5%. The test value exhibited excellent agreement with the simulation prediction, and the average removal rate reached 94%, satisfying the application requirements. These results affirm the reliability of the MBD-DEM coupled simulation test for the shovel-tooth end-effector. Therefore, the optimal operation parameters of end-effector operation were selected as the combination of the angle of soil penetration of 84°, speed of soil penetration of 9.0 cm/s, depth of soil penetration of 8.0 cm and speed of shovel-tooth gathering of 6.0 cm/s.

**Table 7:** Test verification result.

No.	Soil penetration force /N	Shovel-tooth gathering force /N	Removal rate /%
1	34.8	764.5	96
2	35.0	748.2	92
3	34.5	776.3	94
Test average	34.8	763.0	94
Simulation prediction value	33.8	738.5	
Relative error	2.9	3.3	

## Discussion

Hybrid rapeseed has excellent production performance compared with conventional rapeseed, and the demand for seeds is increasing. However, the presence of abnormal plants in the breeding field can negatively impact seed purity and yield. At present, There is no research specifically on machinery for pulling abnormal plants in breeding rape fields. In view of this situation, this paper designed a shovel-tooth end-effector, and built a shovel-tooth removal test bench based on the operation mode of the end-effector. The test bench exhibited a straightforward yet robust structure, ensuring reliable and efficient operation. In this paper, the simulation model was established by MBD-DEM coupling method, and the operation mode of shovel-tooth end effector was simulated. The Design-Expert software was used for analyzing the influence of various test factors on the soil penetration force and the shovel-tooth gathering force and the optimal combination of working parameters to find the lowest operating power consumption. Finally, the end-effector and test bench were fabricated, followed by a field test to validate the operational performance and effectiveness of the shovel-tooth end-effector. The field tests have demonstrated that the shovel-tooth end effector possesses a structurally sound design and effectively fulfills the designated requirements. In practical applications, the device can be mounted on a tractor or other mobile platform. Because the number of abnormal plants in the field is so small, the device is designed to work intermittently, not continuously. First the tractor or other mobile platform moves to the operating position and then the shovel-tooth end-effector performs operation.

This study has carried out some innovative structural design, which has some significance and limitations, as follows:

1. In this paper, we designed a shovel-tooth end-effector for abnormal rape removal. It can replace manual operation, significantly reduce labor costs, and enhance removal efficiency to a certain extent. Moreover, it can also provide a theoretical framework for the research and enhancement of the breeding rape hybrid removal device. The simulation calculation approach presented in this paper is transferable to other crop studies.
2. Because the adhesion force and friction force between rape roots and soil were much smaller than the force of soil extrusion, in the simulation study of this paper, rape plant model was simplified. In the follow-up study, the effect of the presence of rhizomes on the removal of hybrid plants can be considered.
3. In addition, the adaptability and dependability of the end-effector in different field environments should also be considered. In this study, simulation test and field test of yellow clay soil in Nanjing, China were carried out. The prototype is currently in the experimental phase and has essentially fulfilled the design requirements. In the next step, experimental research and structural optimization should be carried out for soil with different characteristics to improve the working ability and stability of the equipment.
4. At present, manual removal of abnormal plants is still the main method in rape breeding fields at home and abroad. In the future, we can increase the research on the removal device of abnormal plants. The shovel-tooth end-effector designed in this paper is large in size. The next step can be to refine and simplify the end-effector. For instance, a more miniaturized apple picking manipulator can be referenced. Furthermore, it can with the robot platform and leverage intelligent technology to enable real-time monitoring of plant growth and accurate identification of plant species, thereby facilitating automated and unmanned operations.

## Conclusion

1. Combined with the growth and agronomic requirements of breeding rapeseed before the initial flowering stage, a shovel-tooth end-effector for the removal of hybrid plants was designed, and a test bench was built.
2. A simulation model based on MBD-DEM coupling was established. We conducted a Box-Behnken test with four factors and three levels. Taking the angle of soil penetration, speed of soil penetration, depth of soil penetration and speed of shovel-tooth gathering as the test factors, the soil penetration force and shovel-tooth gathering force as the test indicators. The influence and interaction of each factor on the response index were determined, and the regression model was established. The optimal operation parameter combination was obtained to reduce the power consumption. A set of optimal parameters was derived based on the established regression equation: the angle of soil penetration of 84°, speed of soil penetration of 9.0 cm/s, depth of soil penetration of 8.0 cm and speed of shovel-tooth gathering of 6.0 cm/s.
3. Based on the field verification test, the average soil penetration force of the three groups of pullout tests was 34.8 N and the average shovel-tooth gathering force was 763.0 N. The relative error of force was less than 5%, demonstrating excellent agreement between experimental and simulation prediction values. The average removal rate was 94%, meeting application requirements, indicating that the simulation test of the shovel-tooth end-effector based on MBD-DEM coupling was reliable.

## Supporting information

S1 Table (DOCX)
