# Supplementary material for: Design and experiment of a shovel-tooth removal end-effector for abnormal plants in hybrid rape breeding based on MBD-DEM coupling
Source: PLoS One. 2023 Dec 14;18(12):e0294919. doi: 10.1371/journal.pone.0294919 (PMC10721175; doi:10.1371/journal.pone.0294919)
Supplement: S1 Table — (DOCX) [file pone.0294919.s001.docx]

**S1 Table.**

| **No.** | **Angle of soil penetration *X*_1_ /**° | **Speed of soil penetration**  ***X*_2_ /(cm·s^-1^)** | **Depth of soil penetration *X*_3_ /cm** | **Speed of shovel-tooth gathering *X*_4_ /(cm·s^-1^)** | **Soil penetration force *Y*_1_ /N** | **Shovel-tooth gathering force *Y*_2_ /N** |
| --- | --- | --- | --- | --- | --- | --- |
| 1 | 84 | 8 | 12 | 8 | 40.35 | 871.12 |
| 2 | 90 | 8 | 12 | 8 | 55.22 | 1085.25 |
| 3 | 84 | 12 | 12 | 8 | 52.31 | 865.30 |
| 4 | 90 | 12 | 12 | 8 | 59.02 | 1075.42 |
| 5 | 87 | 10 | 10 | 6 | 41.13 | 802.15 |
| 6 | 87 | 10 | 14 | 6 | 58.75 | 1075.60 |
| 7 | 87 | 10 | 10 | 10 | 41.03 | 842.00 |
| 8 | 87 | 10 | 14 | 10 | 59.00 | 1108.41 |
| 9 | 84 | 10 | 12 | 6 | 47.02 | 850.18 |
| 10 | 90 | 10 | 12 | 6 | 59.23 | 1065.32 |
| 11 | 84 | 10 | 12 | 10 | 46.56 | 889.48 |
| 12 | 90 | 10 | 12 | 10 | 60.18 | 1100.20 |
| 13 | 87 | 8 | 10 | 8 | 36.45 | 828.61 |
| 14 | 87 | 12 | 10 | 8 | 44.20 | 832.18 |
| 15 | 87 | 8 | 14 | 8 | 53.89 | 1092.10 |
| 16 | 87 | 12 | 14 | 8 | 63.68 | 1081.50 |
| 17 | 84 | 10 | 10 | 8 | 38.25 | 737.14 |
| 18 | 90 | 10 | 10 | 8 | 43.68 | 878.74 |
| 19 | 84 | 10 | 14 | 8 | 51.34 | 1008.81 |
| 20 | 90 | 10 | 14 | 8 | 63.89 | 1268.60 |
| 21 | 87 | 8 | 12 | 6 | 48.00 | 911.22 |
| 22 | 87 | 12 | 12 | 6 | 55.09 | 905.13 |
| 23 | 87 | 8 | 12 | 10 | 47.95 | 988.70 |
| 24 | 87 | 12 | 12 | 10 | 54.95 | 959.26 |
| 25 | 87 | 10 | 12 | 8 | 55.32 | 930.30 |
| 26 | 87 | 10 | 12 | 8 | 54.90 | 932.00 |
| 27 | 87 | 10 | 12 | 8 | 56.20 | 962.68 |
| 28 | 87 | 10 | 12 | 8 | 55.23 | 943.40 |
| 29 | 87 | 10 | 12 | 8 | 51.20 | 926.00 |
